# Genus for biomolecules

**DOI:** 10.1093/nar/gkz845

**Published:** 2019-10-04

**Authors:** Paweł Rubach, Sebastian Zajac, Borys Jastrzebski, Joanna I Sulkowska, Piotr Sułkowski

**Affiliations:** 1 Centre of New Technologies, University of Warsaw, Banacha 2c, 02-097 Warsaw, Poland; 2 Warsaw School of Economics, Al. Niepodległości 162, 02-554 Warsaw, Poland; 3 Faculty of Chemistry, University of Warsaw, Pasteura 1, 02-093 Warsaw, Poland; 4 Faculty of Physics, University of Warsaw, Pasteura 5, 02-093 Warsaw, Poland; 5 Walter Burke Institute for Theoretical Physics, California Institute of Technology, Pasadena, CA 91125, USA

## Abstract

The ‘Genus for biomolecules’ database (http://genus.fuw.edu.pl) collects information about topological structure and complexity of proteins and RNA chains, which is captured by the genus of a given chain and its subchains. For each biomolecule, this information is shown in the form of a genus trace plot, as well as a genus matrix diagram. We assemble such information for all and RNA structures deposited in the Protein Data Bank (PDB). This database presents also various statistics and extensive information about the biological function of the analyzed biomolecules. The database is regularly self-updating, once new structures are deposited in the PDB. Moreover, users can analyze their own structures.

## INTRODUCTION

Biomolecules are often characterized by their primary, secondary, or tertiary structures, which describe the sequence of their fundamental constituents (such as nucleotides or amino acids), and their configuration in physical space. However, in recent years it has been realized that additional non-trivial information about biomolecules, which also characterizes their physical and biological properties, is captured by their (mathematically understood) topology. The topology of a given biomolecule depends not only on local data (such as geometric configuration of a piece of the backbond chain), but on global data (configuration of the whole chain, long-distance interactions, etc.). For example, DNA structures and proteins can be knotted. Other entangled structures found in recent years involve slipknots, links, and lassos. While visual identification of such structures is very hard, in recent years new theoretical and experimental tools have been developed that enable their investigation (1,2). Furthermore, entangled protein chains of the types mentioned above are deposited in databases such as KnotProt (3,4), LinkProt (5) or LassoProt (6). Currently a direction of research devoted to the topological properties of biomolecules is being very actively developed.

The database ‘Genus for biomolecules’ that we present in this work (and which we also refer to simply as the Genus database) assembles information about another important topological property, which is encoded in the genus of a given biomolecular chain or its various subchains. For all proteins and RNA chains deposited in the Protein Data Bank (PDB) (7), we compute the genus trace and genus fingerprint (whose definitions are given in what follows) and other data, and deposit them in the Genus database. The information encoded in such genus characteristics captures, among others, the complexity of bonds in biomolecules (such as base pairs for RNA, or contacts for proteins), the complexity of pseudoknots in RNA, the stability and multi-domain structure of biomolecules.

### Genus characteristics

**Figure 1.**
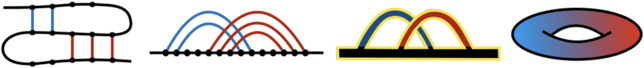
How to compute the genus. A biomolecule (schematically shown in the left, with residues represented by black dots, and bonds by blue and red segments) can be represented by a chord diagram shown as the second item. A chord diagram consists of *b* backbones (horizontal segments) and *n* chords (arcs). Thickening all backbones and chords (and replacing each stack of parallel chords by a single chord or ribbon) gives rise to a ribbon diagram (the third item), which has *r* boundary components (shown in yellow). Such a ribbon diagram can be drawn smoothly on a surface (shown on the right) of genus *g* (the number of ‘holes’) given by the Euler formula *b* − *n* = 2 − 2*g* − *r*. The ribbon diagram in this figure has *b* = 1, *n* = 2, and *r* = 1 (there is one yellow boundary), so that we find *g* = 1.

The genus is a number that is associated with a two-dimensional surface, or equivalently with a chord diagram. In the context of biomolecules it is of advantage to consider the latter interpretation of chord diagrams. A biomolecule—shown schematically in Figure 1 (left), with bonds in blue and red—can be represented as a chord diagram shown as the second (from the left) item in this figure. In general, a chord diagram consists of a number *b* of horizontal intervals (backbones), and *n* chords (half-circles) whose ends lie on those backbones. Each backbone represents one biomolecular chain (so for a single biomolecule it is sufficient to consider one backbone, *b* = 1), and each chord represents one bond (a base pair for RNA, or a contact for proteins). Chords may represent any bonds, and may connect main-chain and main-chain, two side-chains, or side- and main-chain. Chord diagrams are commonly used to encode the structure of base pairs for RNA, and we present the structure of contacts in proteins analogously. For each structure in the Genus database we also draw its chord diagram (in addition taking into account and denoting types of bonds).

To compute a genus of a given chord diagram, one should replace each of its backbones and chords by a ribbon of a non-zero width, as in the third (from the left) item in Figure 1. Note that a stack of parallel chords contributes to the genus the same as a single chord (so each such stack can be replaced by one piece of ribbon). One then obtains the so called ribbon graph, which has *r* independent (one-dimensional) boundaries (shown in yellow in the example in Figure 1). One can then compute the genus *g* assigned to the chord diagram using the Euler relation

(1) $$\begin{equation*} b-n = 2 - 2g - r. \end{equation*}$$

Note that a chord which does not intersect any other chord in a chord diagram does not contribute to the genus. Therefore non-zero values of the genus arise from intersecting chords, and in this sense the genus measures the complexity of bonds. The value of genus is higher, if the pattern of bonds in a given chain is more entangled; for example, the genus for RNA is higher for structures which have more complicated pseudoknots. The genus can thus be regarded as a properly defined quantitative description of complexity of pseudoknots and various biomolecular bonds. The genus also has the interpretation of the number of ‘holes’ of a two-dimensional surface, on which the above mentioned ribbon graph can be drawn smoothly. Such a surface is shown in Figure 1 in the right; the relation (1) implies it has genus *g* = 1. The definition of genus is also presented in the help (‘Read more’) section of our database.

The genus of the whole chain was used to characterize RNA structures, e.g. in (8–15), and to characterize proteins in (16). Furthermore, the relation to two-dimensional surfaces mentioned above makes contact with random matrix theory, and taking advantage of this relation, various approaches to the classification of chord diagrams and pseudoknots have been developed in the above mentioned papers.

For completeness, we recall that bonds that we refer to above (or chords in chord diagrams), are identified with base pairs in case of RNA. In what follows, when considering the genus trace, we also consider various specific types of base pairs, and take advantage of the Leontis–Westhof classification (17,18), which we also summarize below. On the other hand, in case of proteins, we identify bonds (or chords) with contacts, determined as hydrogen bonds between amino acids. Such hydrogen bonds are identified based on atom types and geometrical criteria described in (19); in our analysis we use the tolerance of 0.4 Å in distance and 20° in angle. To identify these bonds we used the function *findhbonds* implemented in Chimera (20).

### Genus trace

In original applications, in particular, in papers mentioned above, the genus was computed only for the entire chain length. However, in (21) a novel characteristic has been introduced, which was called the *genus trace*. The genus trace is a function that encodes the genus of a segment of the biomolecular chain, spanned between the first and the *n*’th residue. It is shown in (21) that the genus trace captures much more information than the genus computed only for the entire chain. In particular, the genus trace enables to visualize, from the viewpoint of the synthesis end, how a molecule folds up during synthesis. The genus trace is calculated and drawn for each structure in our Genus database.

Furthermore, it is shown in (21) that the genus trace can be computed independently for various types of base pairs, both Watson–Crick and non-canonical ones (in case of RNA); this provides yet more accurate characteristics of the biomolecular complexity. Indeed, for RNA we take advantage of the Leontis–Westhof classification (17,18). In this classification each RNA base is treated as a triangle, whose edges are called Hoogsteen (denoted HG or H), Watson–Crick (denoted WC or W), and Sugar or Shallow Groove (denoted S or SG). Base pairs are then formed by glueing two edges that belong to two nucleotides; moreover such edges can be glued in configuration *trans* (t) or *cis* (c). It then follows that 12 types of base pairs can be formed, denoted

(2) $$\begin{eqnarray*} &\rm{cWW,\,tHS,\,tWH,\,tSS,\,cWS,\,tWS,}\nonumber\\ &\rm{cHS,\,tWW,\,cWH,\,tHH,\,cSS,\,cHH.} \end{eqnarray*}$$

These types are ordered by the frequency of their occurrence (22), with the canonical ones (cWW) occurring most often. Genus traces for RNA in our database are drawn taking into account all these types of base pairs (the first plot takes into account only cWW pairs, the second one both cWW and tHS, the third one cWW and tHS and tWH, etc.).

**Figure 2.**
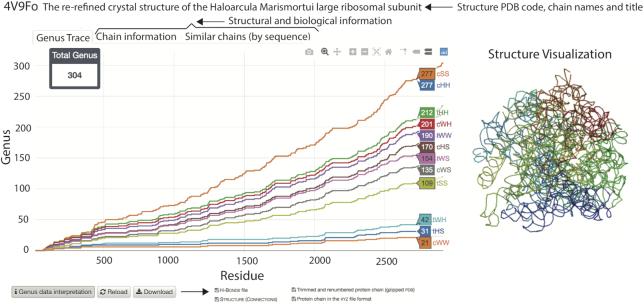
Genus traces for RNA structure—a large ribosomal unit from *Haloarcula marismortui* (PDB code: 4v9f_o_)—together with some other data shown in the database. Genus traces, for various types of base pairs in the Leontis–Westhof classification (17,18) (denoted cWW, tHS, tWH, etc., as also explained in the main text below), are shown in the main diagram (in the left)—the bottom plot (in orange) is the genus trace calculated by taking into account only canonical cWW base pairs; each other consecutive plot includes non-canonical base pairs of an additional type, in the order given in (2); for each genus trace, the genus value for a choice of a residue on the horizontal axis is shown in the colored box. In the top, the PDB ID and the name/title of the structure are shown. Apart from the genus data, one can also view chain information, and a list of similar chains (as indicated by the tabs in the figure). Also the JSmol visualization is shown in the database (right). In the bottom of the figure, some other options and files that can be downloaded, available in the database, are shown.

As an example, genus traces for a large ribosomal unit from *Haloarcula marismortui* (PDB code: 4v9f), together with other data presented in the Genus database for a given structure, are shown in Figure 2.

### Genus fingerprint matrix

**Figure 3.**
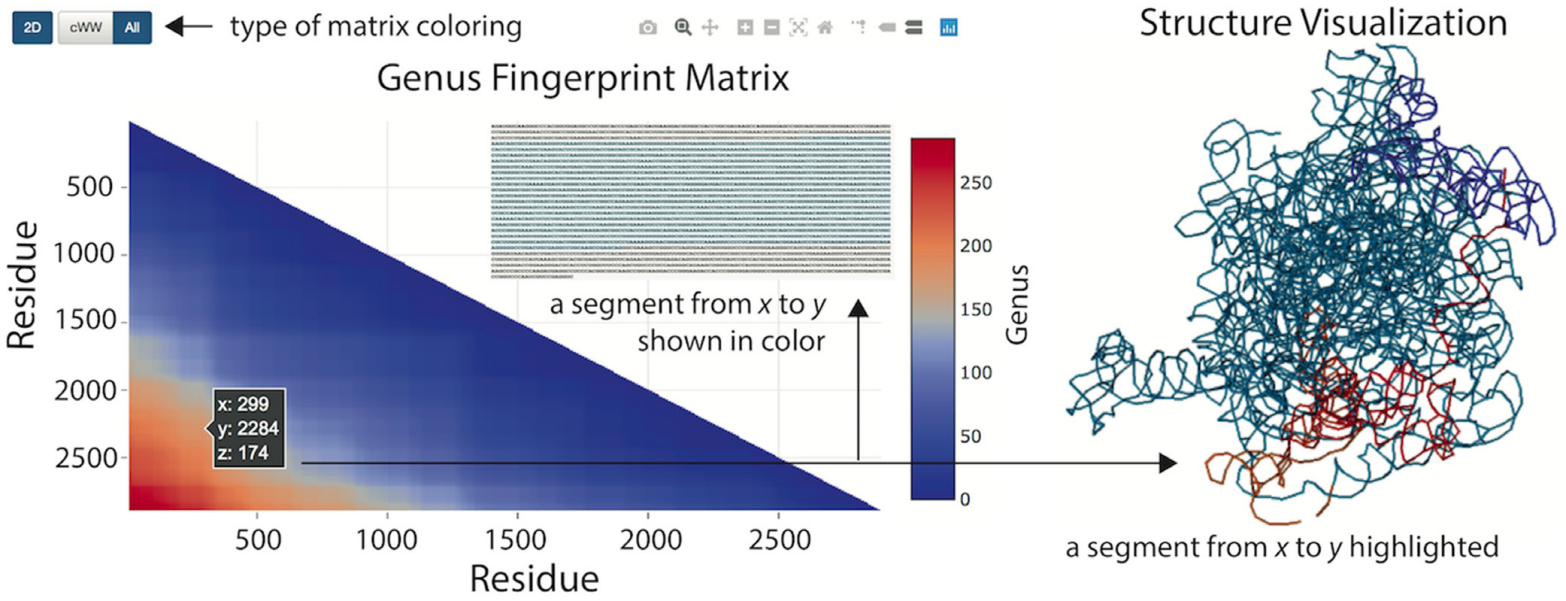
The genus fingerprint matrix a ribosomal subunit (PDB code: 4v5k_BA_), together with some other data shown in the database. The genus matrix is shown in left. At a given point at position (*x*, *y*) (with *y* measured from the top of the diagram), the genus value for a segment of the main chain from the residue *x* to the residue *y* is shown. This genus value is shown by appropriate color (between blue for genus zero, and red for maximal genus), and also by the $z$-coordinate. The user may choose to see the matrix plot in a two-dimensional (as in this figure) or a 3D representation. For RNA chains (as in this case), one can also choose to show canonical base pairs (cWW) only. After clicking a given point in the matrix plot, the corresponding segment is highlighted in color in the sequential representation and JSmol visualization, which are also presented by the database.

In the Genus database we introduce yet more general characteristics, which we call the *genus fingerprint*. Namely, we calculate the genus of each segment of a given chain, spanned between *x*’th and *y*’th residue, and then present these values in a matrix plot. In this plot a point at location (*x*, *y*) (where *y* is measured from the top of the matrix diagram) is shown in an appropriate color, which represents the genus value; these colors change from blue (for zero genus) to red (the highest genus). This matrix plot is what we call the genus fingerprint. An example of a genus matrix for a ribosomal subunit (PDB code: 4v5k_BA_), together with other data presented in the Genus database, is shown in Figure 3. Note that in the database we show fingerprint matrices in two versions: either two-dimensional (where the value of genus is shown in appropriate color), or three-dimensional (where the value of genus is shown in color, and it is also equal to the height above the *x*−*y* plane, i.e. the $z$-coordinate).

The genus fingerprint matrix captures a lot of information (and much more than the genus trace). In particular, it enables to identify segments, of various length, of the whole chain, which contribute most or least to the total genus. Those segments that contribute most have the most entangled pattern of bonds, and should be responsible for specific functions of a biomolecule in question. On the other hand, segments that contribute the least indicate where boundaries of domains are located. In the fingerprint matrix, all points on a given line, parallel to (and below) the diagonal, show how much various segments of the same length (equal to the distance to the diagonal) contribute to the total genus. For example, all points on the diagonal represent segments of zero length, whose genus must be equal to zero, and thus all these points are shown in blue (and so the whole diagonal is shown in blue). On the other hand, the point in the bottom-left corner of the matrix diagram is shown in red, because it represents the whole chain (whose genus is obviously maximal). Therefore, if all bonds would be uniformly distributed, then all lines parallel to the diagonal would have fixed color, and the fingerprint matrix would have triangle-like structure, changing from blue to red once we move from the diagonal to the bottom-left corner (as roughly seen in the example in Figure 3). A deviation from such a triangle-like structure is a manifestation of some particular, non-uniform pattern of bonds, which encodes entangled structure of those bonds and some specific properties of a given biomolecule.

### Structures in the database

The Genus database assembles all biomolecules from the PDB database (7), including crystal, NMR, and EM structures, including those with missing residues. When some residues are missing we can still compute the genus; however, its value can be smaller than the real one, in case some bonds would end along the missing subchain. In case of RNA, we also take advantage of the data presented in the BGSU RNA Site, http://rna.bgsu.edu/rna3dhub/ (23). All structures in our database are available as a list of entries. The database self-updates every Wednesday.

### Database technicalities

The database is written in Python 3 with the Flask framework dynamically generating HTML pages using Apache2 with WSGI. The data is stored using MySQL and Mongo databases. Information about proteins is downloaded from the PDB (7) using RESTful services (in CIF or PDB format), and the Pfam (24) and EC (25) data using the SIFTS service. The identification of hydrogen bonds in proteins is done using UCSF Chimera (20). In case of RNA lists of bonds used in the analysis are downloaded from the BGSU RNA Site database, http://rna.bgsu.edu/rna3dhub/ (23). The genus computation algorithm is implemented in Python 3 and accelerated using Cython. The whole service is installed on multicore Linux nodes and uses an asynchronous message queue (Python Celery) for a reliable and efficient distribution of computing tasks to all compute nodes.

Furthermore, the Genus database is integrated with the KnotProt 2.0 database (4)—on the one hand, information about the presence and type of knots or slipknots for a given protein chain is presented in a table below the genus trace diagram, on the other hand, the KnotProt 2.0 database displays the value of the total genus for every knotted protein chain in the right top corner of the details screen.

The Genus database offers a RESTful API that allows users to run custom queries and download results in a raw textual format or retrieve a full list of all protein and RNA chains with the value of the total genus.

### Database interface

The Genus database is easy and intuitive to navigate. In the homepage, or from the menu on top of each screen, a user can choose to browse database, search database, or process an uploaded structure. One can also simply type a PDB ID of a given structure in the top-right window to view its data.

After choosing the ‘browse’ option, all structures from the PDB (7) are listed; a user can choose to browse through protein or RNA structures. As of beginning of August 2019, there are 23 9574 unique protein chains and 1335 RNA chains deposited in our database. After choosing any of these structures, its genus characteristics are shown in a separate screen, in a way which is summarized below.

In the ‘search’ window there are several options to view the analyzed structures. In the ‘genus’ section all structures are grouped according to their total genus and chain lengths, as shown by corresponding histograms. An interesting plot presenting the dependence of genus on the chain length is also shown. All these histograms and plots are interactive and enable users to choose a group of structures of the user’s interest. Furthermore, in other sections of the database website the user can identify structures of his/her interest based on molecule keywords and molecule tags, and (for proteins) Pfam (24), EC (25) and CATH (26) classification. Finally, an interactive keywords cloud is included. All these classifications are presented separately for protein and RNA structures, and the user can choose to view either all, or only non-redundant structures.

After choosing a biomolecule of interest in one of the above ways, its details and genus data are shown in a separate window. In the first section the genus trace, and genus fingerprint matrix (defined in the previous section) in 2D or 3D representation, are shown as interactive plots, together with a table which presents the total genus and other basic characteristics of a given chain. This and other data presented in the database are also shown in Figures 2 and 3. In case of RNA, we plot genus traces for canonical and various choices of non-canonical base pairs, in the ordering given by (2). Below, a structure visualization in JSmol is shown, which is integrated with with the fingerprint matrix—namely, after clicking a point in the matrix plot, the corresponding subchain is highlighted in JSmol. Finally, a chord diagram for the analyzed structure is shown; in this diagram the backbone chain is represented by a circle, and chords (arcs) connect those residues that are in contact. Structure elements can be removed from this chord diagram by clicking on their symbols. In case of proteins for which it is possible to identify the secondary structure, symbols are given in the Stride classification (e.g. AH stands for ‘alpha-helix’) (27); EMPTY gathers residues that do not belong to any domain in the protein chain.

For a given structure, in the second section ‘Chain information’ various biological and geometrical details are listed: molecule tags and keywords, Pfam annotations (24) and EC nomenclature (25), its total genus and length. Moreover, in two other sections all similar chains are listed.

More details concerning the database interface, statistics, data formats, etc., are given in the help section ‘Read more’, which can also be chosen from the menu in the top of each screen.

### Server and analysis of uploaded structures

The Genus database, apart from collecting genus characteristics for all structures in the PDB (7), also enables users to upload and analyze their own chains. Such chains can be uploaded in CSV format (in which all bonds, crucial for genus computations, are listed) or Chimera format (20). The results are presented in the same format as in the database, and—after computations are completed—are sent to the user by e-mail. In this way, users can analyze for example some artificial structures or non-biomolecular chains.

## DISCUSSION

The Genus database collects information about the genus characteristics of biomolecular chains deposited in the PDB (7). This is important data of topological character, which provides a quantitative measure of the complexity of biomolecules, the complexity of pseudoknots, etc. Note that topological properties of biomolecules have been very actively studied in recent years in other contexts, and our database and the results it assembles complement various other results and topological characteristics found in this line of research. In particular, our database is integrated with the KnotProt 2.0 database (4); for each knotted protein a button is shown which redirects to the corresponding entry in the KnotProt database, and *vice versa* – the information about the genus of each structure is now presented in the KnotProt database.

Currently (in the beginning of August 2019) the Genus database assembles genus characteristics for 239 574 unique protein chains and 1335 RNA chains, and the total of ∼470 000 of all biomolecular chains. The database is regularly updated each Wednesday.

Among various results following from all the data assembled in the database, let us note first an interesting distribution of the total genus values for all protein chains. There are 3373 structures whose genus is 0, and 17 851 whose genus is between 1 and 9. Most common values of genus are in the range 10–30; there are around 50 000 such structures. Then the number of protein chains with larger values of genus systematically decreases. Ultimately, there are several protein chains for which the genus is around 1000. The highest values of genus found to date are 1004 for the structure of the ryanodine receptor in partially open state (PDB code: 4uweA) and 1003 for X-ray structure of an mtbd truncation mutant of dynein motor domain (PDB code: 3vkgA). For the non-redundant set the distribution of genus values is analogous.

For RNA the distribution of the total genus values is different than for proteins. There are 381 RNA chains with genus equal to 0, and 694 structures for which genus is between 1 and 9 (which are most common genus values), and then 56 structures with genus between 10 and 19. Then there are not many structures with larger values of genus, and their distribution is flat, apart from the increase of the number of structures for genus around 150 (there are 88 structures with genus between 130 and 149).

We note that of particular interest are small and large subunits of ribosomes, which are very long RNA chains (of the length, respectively, of the order of 1500 and 3000 nucleotides), whose total genus is of the order of several hundred. For such high values of the total genus, genus traces look like smooth functions, and they capture much more accurate information than in the case of shorter RNA structures, which have much smaller total genus.

Moreover, we find interesting patterns of genus traces, which encode certain properties of biomolecules. For example, plateaus in their plots indicate the domain structure, which enables an automatic and quantitative identification of such domains in biomolecules, as noted already in (21). Furthermore, for RNA we see various patterns of how genus traces corresponding to different nucleotides are built up, and what are contributions of such different nucleotides to the complexity of pseudoknots. This is particularly important information, because to date we lacked simple and quantitative tools that would enable the analysis of pseudoknots and their complexity.

Yet another interesting result are patterns of genus fingerprint matrices calculated in the database, in particular for proteins, for which the total genus is large enough to result in smooth genus matrices. We find that those matrices have some specific shapes and can be grouped into several categories, whose distribution of color (from blue in the diagonal, to red in the bottom-left corner) is triangle-like, square-like, L-like, and has yet another shape. As we explained before (in the section ‘Genus fingerprint matrix’), a triangle-like structure is a manifestation of a uniform distribution of bonds (as roughly seen in the example in Figure 3). Deviations from such a triangle-like structure (e.g. square-like, or L-like patterns) capture some essential information about the geometry and the properties of biomolecular chains. In view of a very large data set in the database, to conduct a thorough analysis of those fingerprint matrices it is of advantage to use automatic algorithms and image analysis techniques (as such a analysis would go beyond a presentation of data in the database, we plan to conduct it in future work).

### Applications

The Genus database should find various applications in several areas of research.

First, the genus data for proteins from the PDB may provide templates to which newly identified structures can be compared, and properties of such new structures can be more reliably predicted. For example, higher values of genus should indicate larger stability of a given chain, while plateaus in genus traces signal its domain structure.

The genus analysis is also of advantage in designing new, artificial RNA and protein structures, which might have applications e.g. in nano-engineering. For example, devising the pattern of base pairs analogous to some particular real RNA structures should enable us to impose requested properties of artificial RNA chains. Some examples of such designed RNA structures are discussed in (28,29), and designed proteins in (30).

Moreover, the vast amount of genus data should be useful for protein folding simulations and structure prediction. For example, as mentioned above, the genus traces enable us to imagine how the complexity of a biomolecular chain is built up upon its synthesis. Therefore, calculating the genus and genus traces during the folding process should provide new reaction coordinates/descriptors, and in consequence additional means of monitoring how the folding proceeds. It would be also interesting to compare the genus data to the so-called contact order (CO), which was used to characterizable protein folding speed. Furthermore, the analysis of the genus characteristics, and the comparison with those already calculated in our database, will undoubtedly be helpful in predicting the secondary and tertiary structure of new biomolecular chains, as well as their function (that should presumably be correlated with the genus characteristics).

The server option of the Genus database could also have various applications, e.g. it could be used to analyze chromatin organization (31).

Furthermore, in view of the relation between chord diagrams and random matrix theory (8–10,14,15), our database and its server option will be useful for applied mathematicians or statistical physicists. For example, the properties of vast ensembles of chord-like structures can be compared with various theoretical predictions in random matrix theory, or such theoretical predictions can be illustrated in explicit examples.

### Comparison with other databases

To the best of our best knowledge, our database is the only one that collects information about genus characteristics. Moreover, it assembles a very large amount of information, i.e. genus characteristics for all structures deposited in the PDB (7) (∼240 000 unique biomolecular chains), with links to various biological information.

As the calculation of genus requires the analysis of a chord diagram – which our database also shows for each protein and RNA structure – let us mention that there are other tools that present chord diagrams, albeit just for RNA structures, e.g. the BGSU database, http://rna.bgsu.edu/rna3dhub/ (23). However, extracting the genus from a chord diagram is still a non-trivial problem, and we are not aware of any other tool or database that would be able to do that.

We also note, that the genus is an example of a topological invariant, and various other databases present information about other topological and entangled structures in proteins, such as knots, slipknots, links and lassos. These other databases include KnotProt 2.0 (4), LinkProt (5) and LassoProt (6). Even though those entangled structures are not immediately related to the genus, it is also of interest to find correlations between such different topological effects. To this end, we integrated the Genus database with the KnotProt 2.0 database, so that a user can immediately check whether a given protein forms a knot or a slipknot (this information is shown in a table below the genus trace diagram), or what is the genus data for some knotted protein being viewed in the KnotProt (the total genus is now automatically shown in the KnotProt). Understanding such correlations may reveal new functions of entangled structures, some aspects of their evolution, their folding mechanisms, and other interesting features.
